# Sound-Evoked Neural Activity in Normal-Hearing Tinnitus: Effects of Frequency and Stimulated Ear Side

**DOI:** 10.3390/brainsci14060544

**Published:** 2024-05-27

**Authors:** Shahin Safazadeh, Marc Thioux, Remco J. Renken, Pim van Dijk

**Affiliations:** 1 Department of Otorhinolaryngology/Head and Neck Surgery, University of Groningen, University Medical Center Groningen, 9700 RB Groningen, The Netherlands; m.a.thioux@umcg.nl (M.T.); p.van.dijk@umcg.nl (P.v.D.); 2 Graduate School of Medical Sciences (Research School of Behavioral and Cognitive Neurosciences), University of Groningen, 9713 AV Groningen, The Netherlands; r.j.renken@umcg.nl; 3 Cognitive Neuroscience Center, Biomedical Sciences of Cells and Systems, University of Groningen, 9713 AW Groningen, The Netherlands

**Keywords:** tinnitus, hyperacusis, tonotopy, auditory cortices, parahippocampal gyrus

## Abstract

Tinnitus is a common phantom auditory percept believed to be related to plastic changes in the brain due to hearing loss. However, tinnitus can also occur in the absence of any clinical hearing loss. In this case, since there is no hearing loss, the mechanisms that drive plastic changes remain largely enigmatic. Previous studies showed subtle differences in sound-evoked brain activity associated with tinnitus in subjects with tinnitus and otherwise normal hearing, but the results are not consistent across studies. Here, we aimed to investigate these differences using monaural rather than binaural stimuli. Sound-evoked responses were measured using functional magnetic resonance imaging (MRI) in participants with and without tinnitus. All participants had clinically normal audiograms. The stimuli were pure tones with frequencies between 353 and 8000 Hz, presented monaurally. A Principal Component Analysis (PCA) of the response in the auditory cortex revealed no difference in tonotopic organization, which confirmed earlier studies. A GLM analysis showed hyperactivity in the lateral areas of the bilateral auditory cortex. Consistent with the tonotopic map, this hyperactivity mainly occurred in response to low stimulus frequencies. This may be related to hyperacusis. Furthermore, there was an interaction between stimulation side and tinnitus in the parahippocampus. This may reflect an interference between tinnitus and spatial orientation.

## 1. Introduction

Subjective tinnitus is a sound perception that is not related to an acoustic sound source. People suffering from tinnitus typically describe hearing a high-pitched tone or noise, often described as ringing in the ear. However, a wide range of tinnitus sounds have been described. Although tinnitus is often of mild impact, its consequences can be devastating. Tinnitus can lead to depression and anxiety, sleep deprivation, and may severely hinder the ability to participate in social activities or work [1,2]. 

Tinnitus is often associated with hearing loss. Plastic changes associated with hearing loss are believed to lead to tinnitus [3]. Indeed, noise-induced changes to cochlear function in animals lead to central changes in spontaneous neural activity in central auditory structures [4,5,6]. Moreover, tinnitus has been linked to incomplete cortical adaptation to hearing loss [7], or, more generally, a failure to adapt to hearing loss [8]. 

However, tinnitus may also occur in the absence of any documented hearing loss. In such cases, tinnitus does not seem to be associated with changes in the tonotopic map [9]. One study showed enlarged responses to sound in normal-hearing tinnitus subjects compared to controls without tinnitus [10,11]. However, Lanting et al. 2014 found no difference in cortical response. In these studies, bilateral stimuli were used. Thus, the responses measured were the net result of simultaneous stimulation of both ears. For a particular hemisphere, the sound-evoked response will be the net result of the sound stimuli presented at each of the ears. For bilateral stimulation, the sound response of the ear ipsilateral to a brain hemisphere probably contributes more to inhibition, while the sound at the contralateral ear may contribute to excitation [12]. In other words, the response of a hemisphere is a mixture of excitation and inhibition contributions of both ears. Consequently, the experiments with bilateral stimuli may have masked effects related to either inhibition or excitation. Therefore, in this study, we measured monaural tone-evoked response to assess brain activity in greater detail. Also, we used pure tone stimuli at four different frequencies to estimate the tonotopic organization of the auditory cortex. We included two high-frequency stimuli (6 and 8 kHz), as an earlier study [7] only detected significant differences between tinnitus and control subjects for higher frequencies. Thus, the aim of this study was to investigate sound-evoked cortical activity in an understudied population of individuals with tinnitus and clinically normal hearing levels. Our design allowed us to test for potential differences in the tonotopic organization of the auditory cortex as well as the effect of the side of stimulation.

## 2. Materials and Methods

### 2.1. Subjects

Eighteen volunteers (39.8 ± 10.9 years old, 12 males) with subjective continuous tinnitus and twenty healthy volunteers (29.4 ± 8.4 years old, 13 males) were recruited for this investigation. The protocol was approved by the Medical Ethics Committee of the University Medical Center Groningen, all participants gave written informed consent, and the research was conducted in accordance with the principles of the Declaration of Helsinki.

Participants were included if they never had any neurological or psychiatric condition, if there was no contraindication for high-field MRI scanning, and if they had no chronic medical conditions that could affect the measurements (e.g., cardiovascular diseases). Pure-tone thresholds were obtained for both ears at all octave frequencies from 125 Hz to 8000 Hz using a standard audiometry device (AC40). Only participants with pure-tone thresholds below 25 dB SPL for all the tested frequencies were included.

For participants with tinnitus, an additional inclusion criterion was continuous subjective tinnitus for at least 6 months. The participants were asked about the possible etiology of their tinnitus.

Every participant completed the Edinburgh Handedness Inventory (EHI) [13], the Hyperacusis Questionnaire (HQ) [14], and the Hospital Anxiety and Depression Scale (HADS) [15]. Participants with tinnitus also filled in the Tinnitus Handicap Inventory (THI) [16] and the Tinnitus Functionality Index (TFI) [17]. These inventories are consistently used in tinnitus neuroimaging studies [7,18], and their use is recommended in American and European clinical guidelines [19,20]. The tinnitus-related questionnaires serve to assess the severity of tinnitus and its common comorbidities. Additionally, tinnitus subjects adjusted the frequency and loudness of a pure tone such that its pitch and loudness matched those of their tinnitus.

### 2.2. Acoustic Stimuli

In the MRI scan, participants were monaurally stimulated with pure tones of frequencies of 353 Hz, 1000 Hz, 6000 Hz, and 8000 Hz. The loudness of the stimuli was matched to that of a 60 dB SPL tone at 1000 Hz. Each stimulus consisted of a train of thirty 100 ms sine waves, including 10 ms rise time and fall time. Each sine wave alternated with a 100 ms silence, forming a 6 s sound wave. A 5-percent frequency deviation (randomly chosen) was embedded in either the first or the last 3 s of each 6 s sound, and participants were instructed to determine if the frequency change was incremental or decremental by pushing one of the two MRI-compatible buttons inside the scanner bore.

### 2.3. Imaging Paradigm

**Figure 1:**
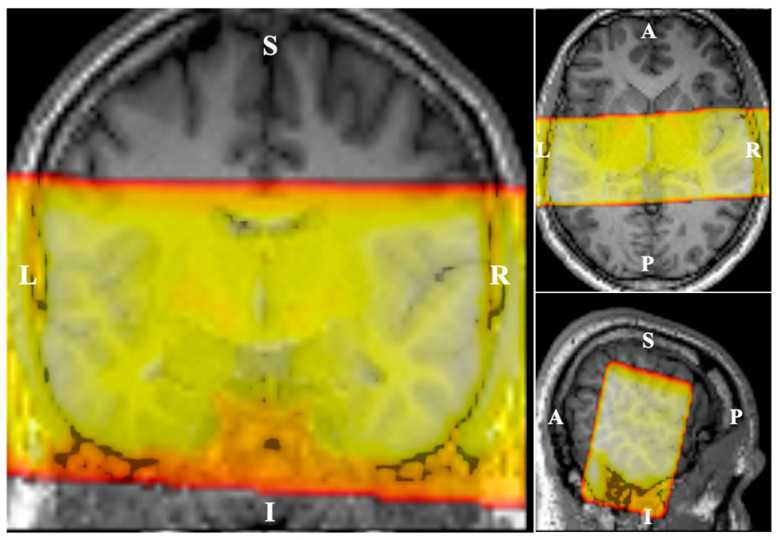
Field of view (FoV) in functional MRI. The fMRI scans were performed coronally with FoV cropped in the superior–inferior direction.

The MRI data were acquired using a 3-Tesla scanner (MAGNETOM Prisma, Siemens Healthineers, Erlangen, Germany) equipped with a 64-channel phased-array head coil. A T1-weighted image (MPRAGE) was acquired for each subject (TR = 2300 ms, TE = 2.98 ms, FoV = 256 mm × 240 mm × 176 mm, 176 slices, 1 mm^3^ isotropic voxel) for anatomical reference. Echo planar imaging (EPI) with reduced field of view in two dimensions (Siemens ZOOMit technique, Figure 1) was used to acquire the functional images (TR = 10 s, TA = 2.97 s, TE = 30 ms, FoV = 192 mm × 84 mm × 176 mm, 29 coronal slices, 2 mm^3^ isotropic voxel). A sparse sampling method (6 s sound stimulation scanner down) was used to prevent interference of scanner noise with the auditory cortex response [21]. For each subject, two runs of functional MRI were performed, 127 brain volumes each. Within one run, each frequency was monaurally presented 14 times to each ear. Additionally, a “silence condition” (no sound played) was presented 15 times, randomly distributed across the full run. A single EPI volume with the opposite phase-encoding direction was acquired after each functional run to allow geometric distortion correction. A T2-weighted image (TR = 1000 ms, TE = 127 ms, FoV = 192 mm × 84 mm × 96 mm, 96 slices, 1 mm^3^ isotropic voxel) with the same FoV as the functional scans was acquired to aid the registration of the functional images to the structural image.

### 2.4. Data Analysis

We used MATLAB (version 2021a) as the main package for processing the data and the SPM12 software package [22] for image registration and functional analysis.

#### 2.4.1. Pre-Processing

As a first step, the potential geometric distortion in the functional images was corrected using the Topup feature from the FMRIB Software Library (FSL) toolbox [23]. The functional volumes were then realigned to the mean volume and registered to the subject’s anatomical image. Then, the functional images were normalized to the Montreal Neurological Institute (MNI) stereotaxic space using the transformation matrix obtained from the anatomical T1 transformation to MNI space. Images were further resampled into a 2 mm × 2 mm × 2 mm isotropic voxel size and smoothed with an isotropic Gaussian kernel with a full-width half-maximum of 8 mm. A log transform was applied to the voxel values of the smoothed images to de-emphasize outliers and obtain a symmetrical bell-shaped distribution of the responses [9]. A Generalized Linear Model (GLM) with eight regressors was applied at the subject level to obtain the neural-evoked response for each and every stimulus type (i.e., four frequencies, left and right presentation) versus the baseline (silence).

#### 2.4.2. Principal Component Analysis (PCA)

We employed PCA to retrieve the most significant patterns in our data by reducing the dimensionality, a procedure that has been used to obtain the tonotopical organization of the auditory cortex [7,9]. For this purpose, a region of interest (ROI) was generated using a group-level GLM that included all voxels showing a significant response to any pure tone relative to the baseline (F-test across all conditions, *p* < 0.01, FWE corrected, minimum cluster size k = 500). Then, within that ROI, the estimated evoked responses of all subjects for each of the eight conditions were concatenated to form an *n* × 8 matrix, which was subsequently subjected to PCA, where *n* is the product of the number of voxels in the ROI and the number of subjects. In order to compare these components between groups, the first 3 resulting components were then separately fed into another PCA and the loadings on the first two components were compared between groups [7,24].

#### 2.4.3. Generalized Linear Model (GLM) Analysis

We further conducted a fully factorial GLM analysis of the data to explore the effect of tinnitus (between-subject variable) and its interaction with sound frequency and laterality (within-subject variables). Thus, the design matrix included two groups (tinnitus and control subjects), two sides of stimulation (left and right), and four frequencies (353 Hz, 1000 Hz, 6000 Hz, and 8000 Hz). The threshold for the statistical parametric maps was set to *p* = 0.001 with a minimum cluster size of 20 voxels [25].

#### 2.4.4. Effects of Tinnitus Severity and Hyperacusis

To further our understanding of differences in neural evoked response associated with tinnitus, we conducted correlation analyses within the regions, showing a significant effect of groups in the GLM (main effect or interaction). The dependent variable was the median evoked response of each participant within these ROIs. The independent variables were the scores for the THI and TFI questionnaires assessing tinnitus burden (tinnitus group only) and the hyperacusis questionnaire assessing hypersensitivity to sounds (across all participants, tinnitus and control subjects). A Pearson correlation was computed between the evoked response and questionnaire scores.

## 3. Results

### 3.1. Group Descriptions

**Figure 2:**
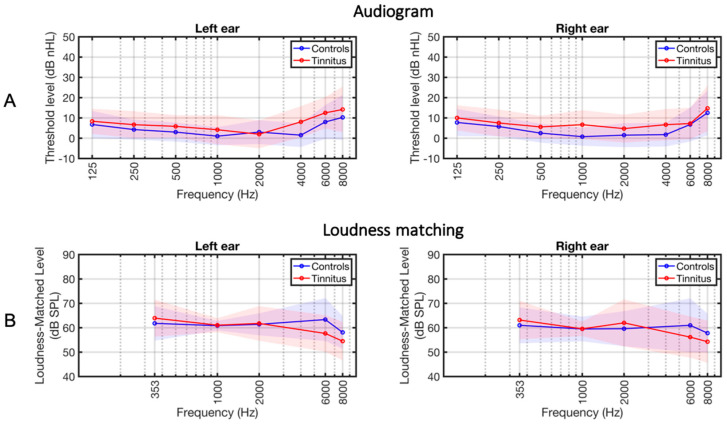
Audiograms and loudness matching. (**A**) Average hearing thresholds of the tinnitus subjects (depicted in red) and the control subjects (represented in blue). The shading indicates the standard deviation per group. (**B**) Average loudness-matched levels. The loudness of tones at frequencies of 353, 1000, 2000, 6000, and 8000 Hz was matched to 60 dB SPL at 1kHz in a randomly selected ear for each subject. During the fMRI experiments, the individually matched tones at 353, 1000, 6000, and 8000 Hz were used for stimulation.

Table 1 summarizes the characteristics of the subject groups. The average thresholds were 7.8 dB HL and 4.8 dB SPL in the tinnitus and control groups, respectively (*t* = 2.26, *p* = 0.029; Figure 2). The intensities of the loudness-matched stimuli, to be used in the MRI experiment, were not different between groups (*t* = 1.32, *p* = 0.18; Figure 2B).

Regarding the etiology of tinnitus, participants’ responses can be classified in a few categories: unknown origin (n = 5), possibly due to loud noise (n = 5), and attributed to stress/emotion (n = 3). Two participants attributed their tinnitus to dental care, one to sleep deprivation during pregnancy, and one reported that tinnitus happened following an episode of dizziness.

The hyperacusis scores were significantly higher in the tinnitus group as compared to the control group (*t* = 3.33, *p* = 0.002). With a cutoff score equal to HQ = 22 [26], the questionnaire indicated the presence of hyperacusis in two controls and nine participants with tinnitus. As indicated in Table 1, the participant groups did not significantly differ with respect to sex, handedness, or HADS score (anxiety and depression). The THI and TFI scores indicated, on average, a moderate tinnitus burden in the tinnitus group. The scores for the HQ, THI, and TFI questionnaires were highly correlated within the tinnitus group (see Supplementary Figure S1).

### 3.2. Principal Component Analysis (PCA)

**Figure 3:**
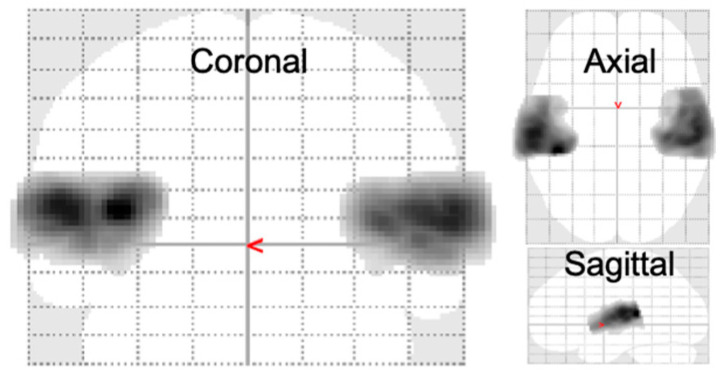
ROIs used in PCA: Significantly sound-evoked voxels in all the subjects. The region-of-interest (ROI) analysis focused on the significantly activated regions resulting from the presented acoustic stimuli (*p* < 0.01, family-wise error (FWE) corrected, cluster size = 500 voxels). In total, 4879 voxels in the left hemisphere and 4230 voxels in the right hemisphere, all located in the superior temporal gyrus, were selected as the ROIs for the subsequent principal component analysis (PCA).

The acoustic stimuli significantly activated a total of 9109 voxels in the left and right auditory cortices (*p* < 0.01, FWE corrected; Figure 3).

**Figure 4:**
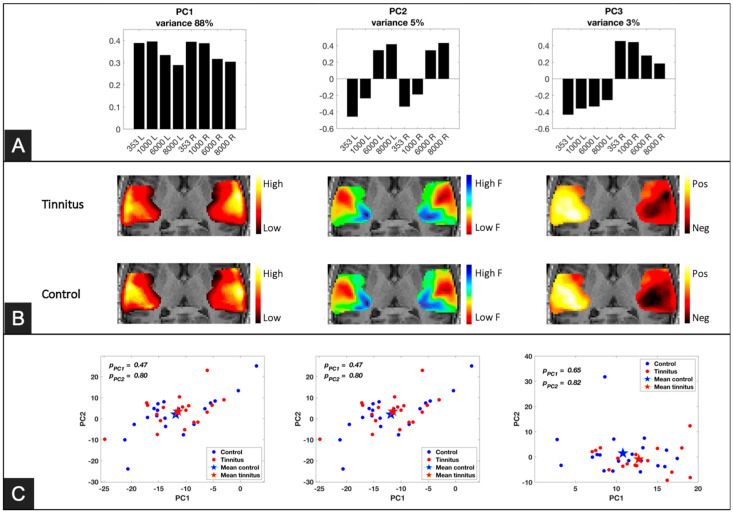
Principal Component Analysis (PCA) results and component maps. (**A**) PCA profiles of the first three components. (**B**) Component maps obtained by projecting each component into the region of interest (ROI). The maps were averaged in the inferior-to-superior direction (that is, perpendicular to the anterior–posterior plane). (**C**) Scatter plots showing the loadings on PC1 and PC2 of the second PCA for each of the three initial PCs.

In the selected region of interest (ROI) representing the auditory cortex, each of the first three principal components corresponded to a distinct aspect of the auditory stimuli (Figure 4). The first component (PC1), accounting for 89% of the total variance, depicted an overall neural response profile to any auditory stimuli. Loadings on that component were relatively higher for low- compared to high-frequency stimuli. The second component (PC2), explaining 5% of the variance, showed a clear staircase profile reflecting the differential neural response to sound frequency. On that component, low-frequency pure tones had negative loadings and high-frequency pure tones had positive loadings irrespective of the stimulated ear. The third component (PC3) classified the laterality of the presented sounds, whereby loadings for left-ear stimulation had a negative sign and those for right-ear stimulation had positive signs. Figure 4A displays the loading profiles of the first three principal components.

The loadings on PC1-3 of each subject were back-projected into the 3D ROIs to generate a response map for each group. The results are depicted in Figure 4 with separate maps for the two groups. The maps for the first component (average response) showed relatively high values in the lateral side of the auditory cortex. These lateral regions are known to encode lower frequencies, and this is reflected in the PC-1 profile showing higher loadings for lower frequencies. The maps of the second component (separating frequencies) showed a typical tonotopic organization of the auditory cortices, where neurons in the posteromedial side of Heschl’s gyrus are dominantly responsive to high-frequency sounds with a gradual V-shape gradient toward the anterolateral side. The maps of the third component (distinguishing the stimulated ear) indicated a clear controlaterality between stimulation side and cortical activity. 

To compare the maps between the groups, we performed another principal component analysis on the maps of each component from the first PCA results (i.e., PC1, PC2, and PC3). The analysis represented the response maps of each subject with just three scores (Figure 4C). This analysis did not show any difference between the means of differences of the scores between the groups.

### 3.3. Full Factorial GLM Analysis

To further explore potential differences between groups, a full factorial GLM analysis was conducted, including group as the between-subject variable as well as tone frequency and stimulation side as within-subject variables. We looked for main effects of group (tinnitus vs. controls), as well as interactions between group and frequency or side of stimulation on the auditory cortex sound-evoked response within the field of view (Figure 1). Statistical maps were thresholded at the voxel level (*p* < 0.001, uncorrected) with a minimum cluster size of 20 voxels.

### 3.4. Main Effect of Group

**Figure 5:**
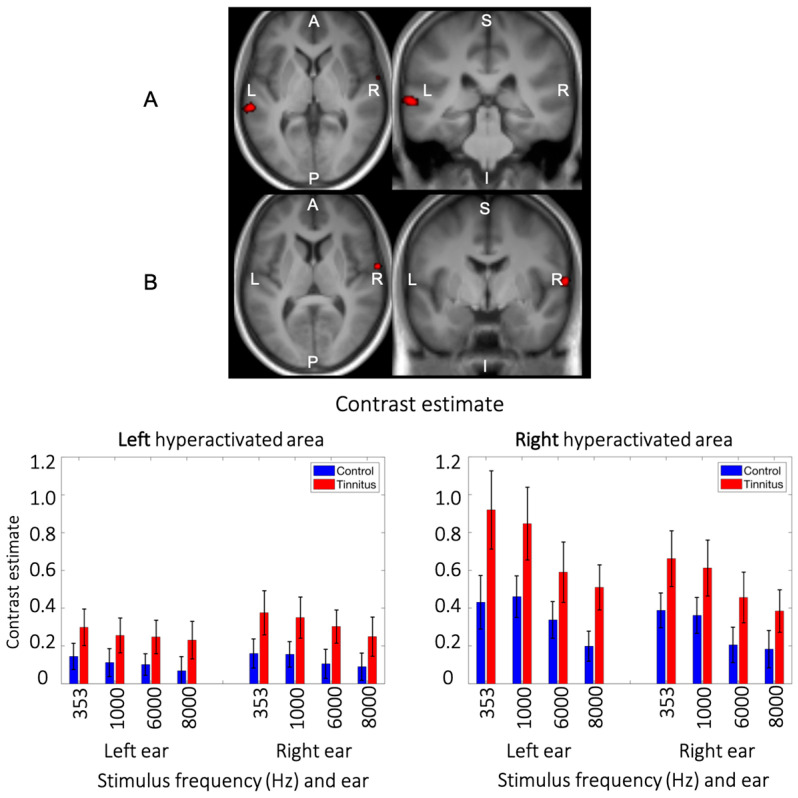
The main effect of tinnitus. Hyperactivated regions in the lateral side of the auditory cortex. Enhanced evoked response (*p* < 0.001, uncorrected) was observed in the posterolateral region (left hemisphere) and the anterolateral region (right hemisphere) of the auditory cortex among subjects with tinnitus compared to controls. The hyperactivated area in the right hemisphere overlaps with the hyperactivated area of the PC1 map in tinnitus ((**B**), left maps). The bar plots indicate the median evoked response to each stimulation, separately in the left and right hyperactivated regions.

A main effect of group was found in two regions of the lateral auditory cortices. The first cluster (55 voxels; MNI peak coordinates: −62, −30, 2; F_1,288_ = 16.86) was located in the left posterior superior temporal gyrus/superior temporal sulcus (Figure 5A). The second cluster (29 voxels; MNI peak coordinates: 64, −4, 8; F_1,288_ = 15.92) was located more anteriorly in the right superior temporal gyrus/Heschl’s gyrus, with some voxels in the Rolandic operculum (Figure 5B). In both regions, participants with tinnitus showed higher evoked auditory response compared to controls, irrespective of pure tone frequency and stimulated ear (Figure 5). The plots of the contrast estimates across all conditions also show a clear staircase profile, where, in these two lateral auditory regions, lower frequencies evoked higher responses than higher ones, in accordance with the tonotopic organization of the auditory cortex (Figure 5). 

We further tested for correlations between brain evoked responses and scores for questionnaires on tinnitus burden (THI and TFI) and hyperacusis (HQ) within these two auditory brain regions, showing significant main effects of group (Supplementary Figure S2). In the tinnitus group, tinnitus burden (THI and TFI) scores were positively associated with the median sound-evoked response for the lowest frequency (353 Hz) only, and more so in the right hemisphere following left-ear stimulation. Similarly, for the hyperacusis scores, a positive correlation with the median evoked response was found solely for the 353 Hz stimulation in the right hemisphere following left-ear stimulation in the tinnitus group.

### 3.5. Interaction between Group and Side of Stimualtion

**Figure 6:**
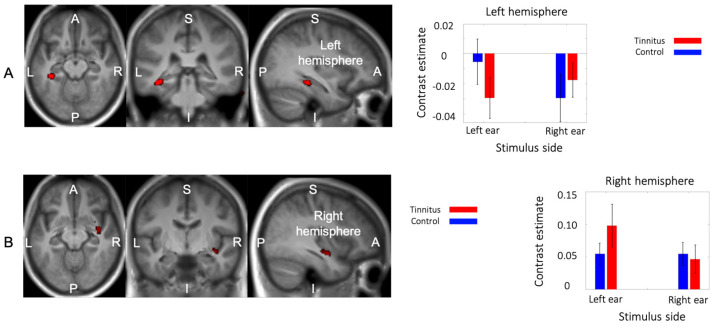
Interaction between group and sound laterality. (**A**) The interaction analysis showed a reduction in activity in the left parahippocampus in response to sound, except in the left ears of control subjects. (**B**) In the right superior temporal gyrus, there was an increase in activity in response to sound, specifically in tinnitus subjects when a stimulus was applied to the left ear.

An interaction between group and the stimulated ear was found in two regions. The first cluster (59 voxels; MNI peak coordinates: −36, −34, −14; F_1,288_ = 20.2) was located in the left parahippocampal region, with a peak in the collateral sulcus in between the parahippocampal and fusiform gyri (Figure 6A). This cluster included seven voxels in the hippocampus proper. The second cluster (34 voxels; MNI peak coordinates: 38, −8, −12; F_1,288_ = 13.22) was found in the right superior temporal gyrus in the vicinity of the lateral fissure and the insular cortex (Figure 6B). 

The contrast estimates of the two groups for the two stimulated ears are also shown in Figure 6. In the left parahippocampal region, the control group showed decreased activity relative to the baseline following right- but not left-ear stimulation. Participants with tinnitus, in contrast, showed decreased activity during sound presentation, irrespective of the stimulated ear. In the right superior temporal cluster, sound presentation was associated with increased BOLD response for both groups and both stimulation sides, but in the tinnitus group the response was higher following contralateral (left-ear) stimulation.

### 3.6. Triple Interaction: Group × Stimulation Side × Frequency

**Figure 7:**
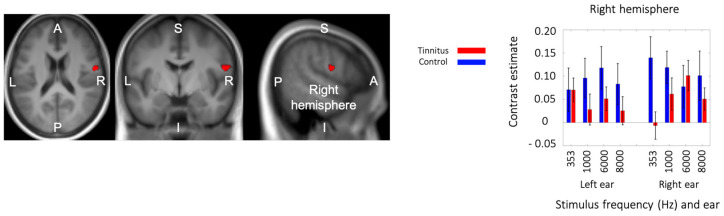
Triple interaction between group, stimulus frequency, and stimulation side in the inferior part of the Rolandic fissure in the right hemisphere. The interaction analysis showed higher activity in the control group, which depended on the stimulus frequency and the stimulated ear.

There was no significant interaction between group and sound frequency; however, there was a significant triple interaction between group, frequency, and stimulation side (Figure 7). The cluster (41 voxels; MNI peak coordinates: 56, −4, 18; F_3,288_ = 7.64) was found in the inferior part of the Rolandic fissure (pre- and postcentral gyri). 

The plot of the contrast estimates for the eight conditions and the two groups (Figure 7) shows that, in the control group, this Rolandic region was significantly active relative to the baseline for all conditions and displayed a relatively strong response to low-frequency (353 Hz) stimuli presented to the right ear. In the tinnitus group, in contrast, this region was not active above the baseline in most of the conditions, with the exception of 4 kHz stimuli presented to the right ear and 353 Hz stimuli presented to the left ear.

## 4. Discussion

This study sought to examine differences in sound-evoked brain response associated with tinnitus in individuals with clinically normal hearing levels. To this effect, tinnitus and control participants were monaurally stimulated with pure tones of four different frequencies. We first applied a Principal Component Analysis (PCA) to the data in order to retrieve the tonotopic organization of the auditory cortex in both groups [27]. Three components accounted for most of the variance in the signals. The first component showed positive loadings for all conditions (especially for low frequencies, 353 Hz and 1 kHz). The second component revealed the tonotopic organization of the auditory cortex. The third component reflected the side of stimulation (left or right ear). In agreement with one previous research [9], we found no significant difference in terms of tonotopic organization of the auditory cortices in the participants with tinnitus and no hearing loss, at least at a macroscopic level. Furthermore, both groups (tinnitus and control subjects) showed a typical pattern of higher cerebral activity contralateral to the stimulated ear [11,28]. These results support the idea that hearing loss, not tinnitus, is the main factor driving functional and neuroanatomical changes in the auditory cortex [3,7,18]. 

Although PCA can provide a data-driven picture of auditory cortex behavior, comparing the maps between groups is challenging. For instance, higher loadings for the first component in the right lateral auditory cortices of the participants with tinnitus was not captured by our between-group analysis. Statistical Parametric Mapping (SPM) was therefore used to fit a full factorial model to the data at the voxel level, testing for the effect of group and its interactions with side of stimulation and tone frequency. This analysis revealed a significant main effect of group in two regions of the lateral auditory cortices (left superior temporal gyrus and right Heschl’s gyrus). Participants with tinnitus showed higher sound-evoked response relative to controls in both regions. These lateral areas also displayed relatively higher responses for sound of lower frequencies in accordance with the tonotopic mapping. Interestingly, the sound-evoked response in these two regions was correlated with tinnitus burden and hyperacusis scores, but only for low frequencies. Because hyperacusis (hypersensitivity to sounds of mild or moderate intensity) was highly correlated with tinnitus burden in our group, it was, however, not possible to disentangle the contribution of these two factors.

Hyperacusis in tinnitus patients is associated with enhanced cortical response to both low- and high-frequency stimuli [29]. In contrast, effects of tinnitus are only present in response to high-frequency tones and primarily in high-frequency portions of the auditory cortex [7]. Together, this suggests that hyperacusis is associated with pathological response across the frequencies and therefore across the tonotopic map, while the pathology of tinnitus appears to be more localized in high-frequency cortical areas. This corresponds to the pitch of tinnitus, which typically corresponds to that of a high-frequency tone. If hyperacusis is indeed associated with pathology across the tonotopic map, the hyperactivity in the lateral portion of Heschl’s gyrus [9] is likely associated with hyperacusis rather than tinnitus. 

Our analysis also revealed an interaction between group and side of stimulation in the parahippocampal cortex. In control subjects, the left parahippocampus showed inhibition in response to sounds presented to the contralateral but not the ipsilateral ear. In participants with tinnitus, the same region was below the baseline for both left- and right-ear stimulation. In other words, left-ear stimulation stood out as deviant from other stimulation conditions between the groups. The parahippocampus has been associated with tinnitus, primarily in resting-state studies. In tinnitus, beta- and gamma-band activity is enhanced [30], and functional connectivity is increased between the parahippocampal cortex and the auditory cortex [31] or more generally with the auditory resting-state network [32]. The parahippocampus was not reported in functional MRI studies that studied sound-evoked response. Here, however, we could show an interaction with the side of stimulation due to our design with monaural stimuli. The parahippocampus is involved in memory retrieval, and it has been suggested that dysfunctional gating of perceptual memory may result in a phantom percept, that is, tinnitus [33]. It seems, however, unlikely that the high-pitched tonal percept of which tinnitus often consists is based on retrieval from memory. On the basis of our results, we confirm a role of the parahippocampus in tinnitus but cannot provide support for the hypothesis that this role concerns sound-memory retrieval. Indeed, our participants with normal hearing thresholds do not need to fill a gap in the high-frequency band. Our results instead seem to suggest an important effect of laterality of sound presentation, which could be related to tinnitus sound localization in a map of the environment or an interference between tinnitus and spatial sound perception [34].

Finally, a triple interaction was found in the right inferior central sulcus, a motor area associated with tongue and lip movements [35]. Control participants showed a positive activity relative to the baseline in this region, with a high response for low-frequency pure tones, in sharp contrast to tinnitus participants who only showed a significant response for high frequencies presented to the right (ipsilateral) ear. 

We found no interaction between group and pure tone frequency, thereby confirming the normal tonotopic organization of the auditory cortex in patients with tinnitus and normal hearing thresholds.

In this study, we only obtained pure-tone audiometry up to 8 kHz, as in clinical practice. However, the groups might diverge at higher frequencies. This is indeed likely to be the case, as participants in the tinnitus group were on average older than those without tinnitus, and age is a main factor explaining hearing loss at high frequencies [36].

Testing very young participants with tinnitus who do or do not present signs of very-high-frequency hearing loss would be important. Nonetheless, high-frequency (>8 kHz) hearing loss is unlikely to explain the differences we found in this study regarding sound stimulation at low frequencies.

## 5. Conclusions

In conclusion, in individuals with tinnitus and clinically normal hearing levels (<25 dB SPL for all frequencies up to 8 kHz), the tonotopical organization of the auditory cortices seems to be preserved. However, these individuals showed elevated sound-evoked responses in two lateral auditory regions. This hyperactivity could be related to hyperacusis rather than tinnitus, but further studies will be needed to clarify the difference between hyperacusis and tinnitus burden with respect to these group effects. Furthermore, we found an effect of the laterality of sound presentation on parahippocampal activity as a function of group. The parahippocampal gyrus has been often associated with tinnitus in resting-state studies. Here, we demonstrate that this region, involved in memory but also spatial navigation, distinguishes between groups following left-ear stimulation in normal-hearing participants.

## Figures and Tables

**Table 1:** Subject characteristics. Averages per group were compared using a *t*-test. The hearing thresholds were measured at the following frequencies: 125, 250, 500, 1000, 2000, 4000, 6000, and 8000 Hz. For sex and handedness, a chi-square test was used to compare the groups (†).

	Tinnitus	Control	*t*-Value	*p*-Value
Age (years old)	40.2 ± 10.5	29.5 ± 8.5	3.4392	0.0015
Sex	12 M, 6 F	13 M, 7 F		0.9139 †
Hearing Threshold dB (nHL)	7.8 ± 3.7	4.8 ± 4.2	2.2650	0.0295
Handedness	3 L, 15 R	5 L, 15 R		0.5292 †
Anxiwty score	6.4 ± 3.8	4.8 ± 3.3	1.3744	0.1778
Depression score	4.5 ± 3.7	3.2 ± 2.6	1.2786	0.209
Hyperacusis score	21 ± 7.4	12.8 ± 7.5	3.3385	0.002
Tinnitus Handicap Inventory	36.8 ± 21.7	-		-
Tinnitus Funcional Index	34.5 ± 19.7	-		-
Tinnitus laterlity	3 L, 1 R, 12 bi	-		-

†: Chi-squared.

## Data Availability

The data presented in this study are available on request from the corresponding author due to ethical reasons.

## Supplementary Materials

The following supporting information can be downloaded at: https://www.mdpi.com/article/10.3390/brainsci14060544/s1, Figure S1: Correlation between the questionnaire scores within tinnitus group; Figure S2: Correlation between the neural activity of the lateral hyperactivated regions of the auditory cortex and questionnaire scores.
