# Supplementary material for: Sound-Evoked Neural Activity in Normal-Hearing Tinnitus: Effects of Frequency and Stimulated Ear Side
Source: Brain Sci. 2024 May 27;14(6):544. doi: 10.3390/brainsci14060544 (PMC11201825; doi:10.3390/brainsci14060544)
Supplement: Supplementary file 1 [file brainsci-14-00544-s001.zip › brainsci-2988429-supplementary.pdf]

Supplementary figures

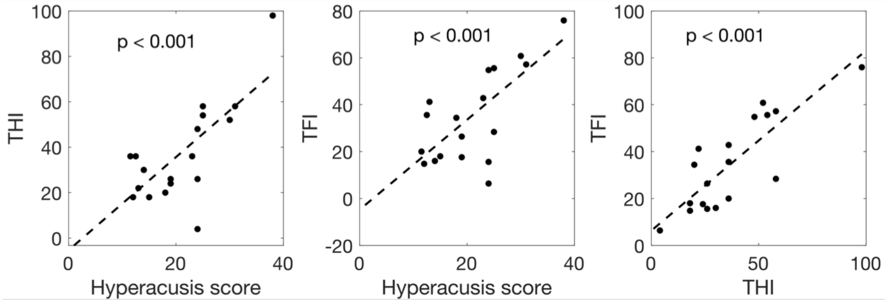

A B C

Supplementary Figure S1. Correlation between questionnaire scores within the tinnitus group. A) Hyperacusis scores in the tinnitus group significantly correlates with the THI scores. B) Hyperacusis scores in the tinnitus group significantly correlates with the TFI scores. C) THI scores in the tinnitus group significantly correlates with the TFI scores.

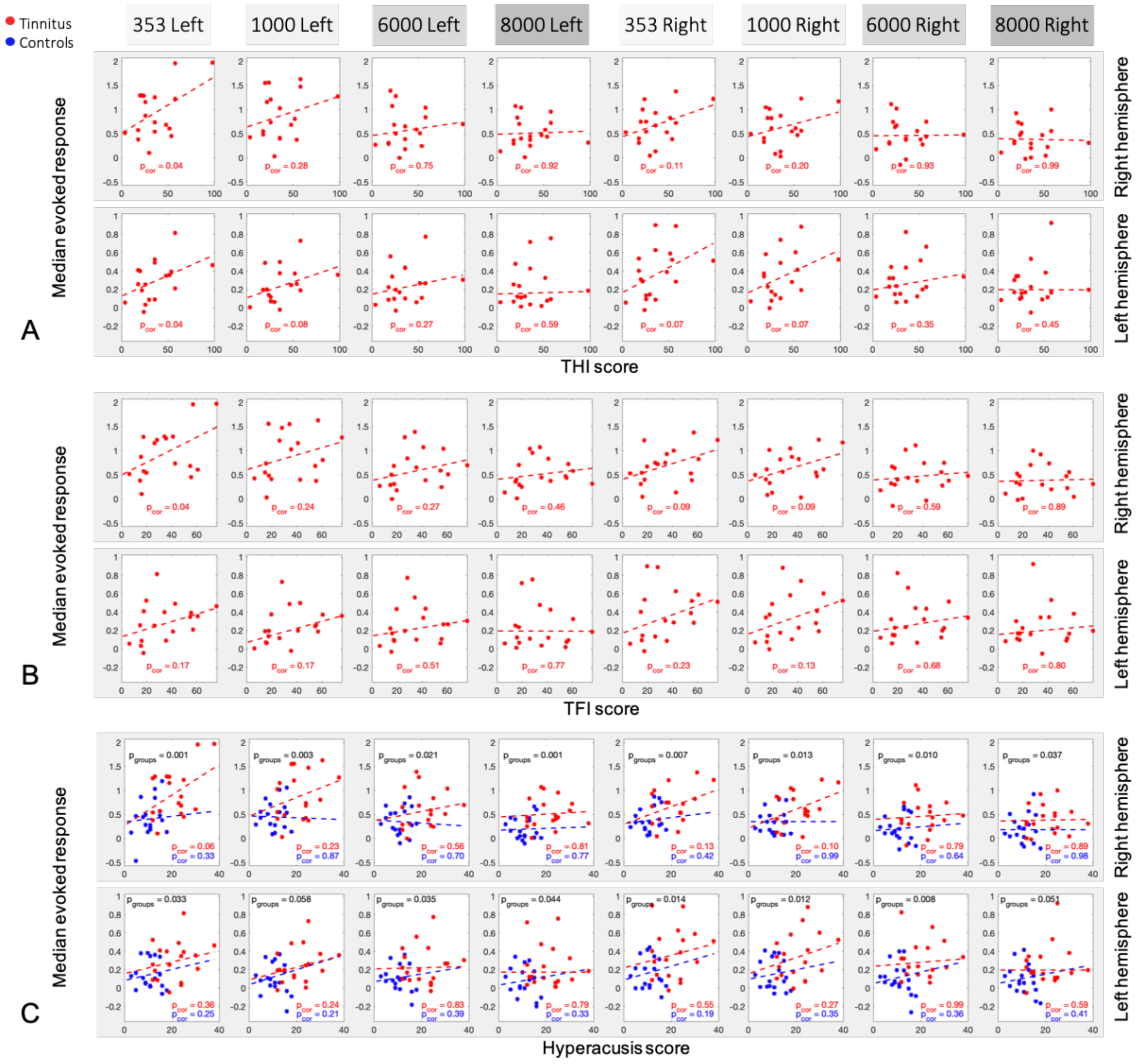

Supplementary Figure S2. The correlation between the neural activity of the lateral hyperactivated regions of the auditory cortex and questionnaire scores. Each panel shows the relation between the neural response amplitude and a questionnaire score, where each individual data point corresponds to one participant. Each column is labeled by the frequency and laterality of the corresponding stimulus condition. A) Tinnitus Handicap Inventory (THI, range 0-100) vs. median evoked response. B) Tinnitus Functional Index (TFI, range 0-100) vs. median evoked response. C) The hyperacusis questionnaire (HQ, range 0-42) vs. median evoked response. In panels A and B, the subjects without tinnitus are not plotted since they did not fill in the THI and TFI questionnaires. In these panels, the horizontal dashed blue line represents the median evoked response of these tinnitus subjects. All other dashed lines (red lines in panels A and B, red and blue lines in panel C) are regression curves to the data point of the Spearman corresponding color. The corresponding p-value indicates the statistical significance of the correlation in that panel. The median evoked response in tinnitus patients was more correlated with tinnitus scores when stimulated with low frequencies (mainly, 252 Hz, and partially, 1000 Hz). This correlation dramatically reduces with raising the stimulation sound frequency. The tinnitus patients showed a similar correlation pattern with the hyperacusis score. However, subjects without tinnitus did not show any correlation between the median evoked response and hyperacusis score.
